# Eriodictyol can modulate cellular auxin gradients to efficiently promote in vitro cotton fibre development

**DOI:** 10.1186/s12870-019-2054-x

**Published:** 2019-10-24

**Authors:** Anam Qadir Khan, Zhonghua Li, Muhammad Mahmood Ahmed, Pengcheng Wang, Xianlong Zhang, Lili Tu

**Affiliations:** 1 National Key Laboratory of Crop Genetic Improvement, Huazhong Agricultural University 430070, Wuhan, Hubei People’s Republic of China; 2 Institute of Plant Breeding & Biotechnology, MNS University of Agriculture, Multan, Pakistan

**Keywords:** Auxin transport, Cotton fibre, Eriodictyol, Fibre development, Flavonoids, Flavonoid biosynthesis

## Abstract

**Background:**

Flavonoids have essential roles in flower pigmentation, fibre development and disease resistance in cotton. Previous studies show that accumulation of naringenin in developing cotton fibres significantly affects fibre growth. This study focused on determining the effects of the flavonoids naringenin, dihydrokaempferol, dihydroquerectin and eriodictyol on fibre development in an in vitro system.

**Results:**

20 μM eriodictyol treatment produced a maximum fibre growth, in terms of fibre length and total fibre units. To gain insight into the associated transcriptional regulatory networks, RNA-seq analysis was performed on eriodictyol-treated elongated fibres, and computational analysis of differentially expressed genes revealed that carbohydrate metabolism and phytohormone signaling pathways were differentially modulated. Eriodictyol treatment also promoted the biosynthesis of quercetin and dihydroquerectin in ovules and elongating fibres through enhanced expression of genes encoding *chalcone isomerase*, *chalcone synthase* and *flavanone 3-hydroxylase*. In addition, auxin biosynthesis and signaling pathway genes were differentially expressed in eriodictyol-driven in vitro fibre elongation. In absence of auxin, eriodictyol predominantly enhanced fibre growth when the localized auxin gradient was disrupted by the auxin transport inhibitor, triiodobenzoic acid.

**Conclusion:**

Eriodictyol was found to significantly enhance fibre development through accumulating and maintaining the temporal auxin gradient in developing unicellular cotton fibres.

## Background

Flavonoids are a diverse group of secondary metabolites that are commonly found in nature and play various roles that assist plants in multiple biological activities [1, 2]. They have been proven beneficial to various crop plants by providing resistance against pathogen infections [3]. However, the most visible impact of flavonoids is their potential to produce pigmentation in flowers, fruits and seeds [4]. Flavonoid biosynthesis leads to the production of six different classes of compounds amongst which the anthocyanins, responsible for pigmentation in flowers, are widespread in plants [5]. In cotton, approximately 52 flavonoids are involved in modulating fibre development, pigmentation and regulating other functions [6]. Color variations in cotton flower petals have also shown to be influenced by flavonoid accumulation [7]. Silencing of *FLAVANONE 3-HYDROXYLASE* (*F3H*; *TT6*) in cotton blocked flavonoid biosynthesis which changed the flower petal color from cream to white, due to reduced anthocyanin content [7].

Flavonoids are considered crucial in modulating pigmentation in naturally colored cotton fibres and recently, many studies have explored the genetic and metabolic basis of fibre pigmentation using biotechnological tools. A study reported that general flavonoid biosynthesis pathway genes, such as *CHALCONE SYNTHASE* (*CHS*), *FLAVANONE 3′-HYDROXYLASE* (*F3’H*) and *FLAVANONE 3′5’-HYDROXYLASE* (*F3’5’H*), were differentially expressed in brown cotton fibres compared to white cotton fibres [8]. Combined transcriptomic and metabolomic analyses showed that proanthocyanins (PAs) such as gallocatechin and catechin were abundantly present in brown cotton fibres because flavonoid biosynthesis pathway genes were highly expressed [9]. Another study reported that a TT2-type MYB transcription factor modulated proanthocynidin biosynthesis through interacting with promoter elements to enhance the transcriptional activity of two downstream flavonoid biosynthesis genes *ANTHOCYANIDIN REDUCTASE* (*ANR*) and *LEUCO-ANTHOCYANIDIN REDUCTASE* (*LAR*) [10]. The overexpression of *GhTT2-3A* during secondary cell wall synthesis resulted in brown cotton fibres [11]. Although flavonoid biosynthesis in pigmented fibres is well established in cotton, engineering of this pathway for the production of higher-quality cotton fibres has not been well exploited.

Over the last decade, many researchers have emphasized the putative role of the flavonoid metabolic pathway in the modulation of anthocyanin and PA biosynthesis in cotton [8, 12]. Interestingly, transcriptomic analyses have suggested a potential involvement of flavonoids in fibre development, since various flavonoid biosynthesis genes have been found to be differentially expressed in developing fibres [13, 14]. Similarly, comparative transcriptome and metabolome analyses have revealed a significant abundance of flavonoids in elongating fibres of *G. barbadense*, a cultivated tetraploid cotton that produces high-quality fibre [15, 16]. However, the critical role of flavonoids during fibre development remained obscure until a study demonstrated that silencing *F3H* suppressed fibre elongation due to the accumulation of naringenin (NAR) [17].

Flavonoids have also been considered to be modulators of auxin signaling since they may be distributed asymmetrically, thus affecting auxin transport by enhancing localized accumulation [18, 19]. They have been demonstrated to interact with auxin transport components through a class of transcription factors, *WRKY*, which ultimately regulate root development in Arabidopsis [20]. Recently, a review summarized compelling evidence that flavonoids mediate phytohormone signaling and growth responses in plants [21]. Flavonoids may modulate auxin signaling and transport through their ability to scavenge reactive oxygen species (ROS) [22] and can affect a wide range of proteins [18]. Although flavonoids were once considered to inhibit auxin signaling, in reality, they establish an auxin gradient in single cells [23]. However, the link among flavonoids, auxin and growth has not been extended to unicellular cotton fibre cells in vitro or *in planta*.

Several developmental processes such as morphogenesis, hormone signaling and ROS homeostasis in the perspective of fibre biology have been studied using cotton ovule culture [17, 24–26]. The critical role of flavonoids in cotton fiber development was also witnessed using the cotton ovule culture. Interestingly, endogenous levels of two flavanols (eriodictyol, ERI and naringenin, NAR), two dihydroflavanols (dihydrokaempferol, DHK and dihydroquerectin, DHQ) and two flavanones (kaempferol, K and quercetin, Q), were found to be influenced by *F3H* activity in the F3H-transgenic cotton lines, and NAR was found to inhibit fibre development [17]. To further dissect the molecular mechanisms underlying the role of flavonoids, we here examined the effects of six different, exogenously applied flavonoids on cotton fibre development. We determined the optimal concentration at which maximal in vitro fibre growth was observed and found that ERI significantly promotes fibre development. RNA-seq analysis was performed to gain insights into the transcriptional networks modulated by ERI treatment that could promote fibre growth in ovule culture. This study suggests the potential of the flavonoid ERI in promoting fibre growth through effects on fibre initiation and elongation.

## Results

### Exogenous ERI promotes fibre development in vitro

**Fig. 1:**
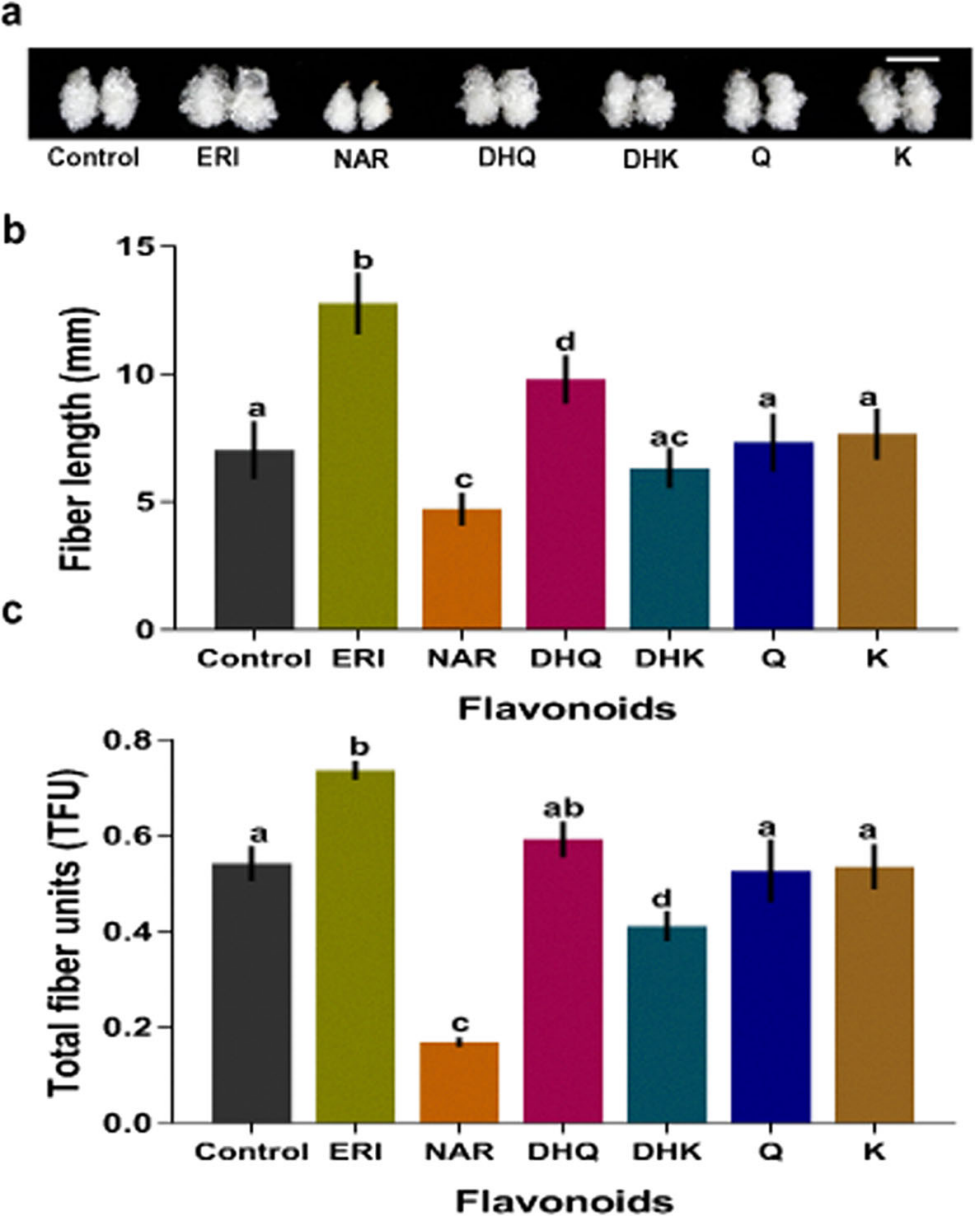
Exogenous ERI treatment promotes in vitro fibre development. (**a**) The fibre phenotype of 10-day-cultured ovules without or with different flavonoids treatments, i.e., control, ERI, NAR, DHQ, DHK, Q and K. The white line represents the *scale bar*, 5 mm. (**b**) Fibre length after 10 days of culture from in response to different flavonoid treatments. (**c**) Total fibre unit (TFU) in 10-day-cultured ovules in response to different flavonoids. For b-c, *error bars* indicate SD for three repeats, and *lowercase* letters present significant differences between the treatments

Genetic engineering of the flavonoid metabolic pathway is considered promising for improving fibre development. Silencing *F3H* leads to NAR accumulation, which is negatively associated with fibre development, while its overexpression does significantly affect fibre phenotype [17]. It is therefore of significant interest to understand the expression pattern of flavonoid pathway genes during in vitro cotton fibre development, and how this is linked to growth effects. An ovule culture assay was performed in which 6 different flavonoids (ERI, NAR, DHQ, DHK, Q and K) were supplemented to the BT medium at a concentration of 10 μM. Fibre development in response to flavonoid treatment was determined by measuring fibre length and the total fibre unit (TFU) compared with controls. After 10 days of culture, the fibre phenotype of cultured ovules was observed to be differentially influenced by the six flavonoids (Fig. 1a). NAR and DHK both suppressed fibre development in terms of fibre length and TFU (Fig. 1b-c). Treatment with two flavanols (Q and K) did not significantly affect fibre development, and therefore were not investigated further (Fig. 1b-c). Effects on fibre length and TFU were investigated following in vitro ovule treatment with ERI and DHQ, both of which are produced through the enzymatic action of F3’H on NAR and DHK respectively (Fig. 1c). ERI efficiently promoted fibre development, which suggests that *F3’H* activity may have a role in determining fibre growth dynamics during early fibre development.

### ERI promotes the initiation and elongation stages of fibre development

In vitro ovule culture assays provided an opportunity to test the effect of flavonoids on fibre development. We determined the optimum ERI concentration needed for maximum fibre growth in vitro and compared the responses of NAR, DHK and DHQ in the presence of growth-regulating hormones, i.e., indole acetic acid (IAA) and gibberellic acid (GA_3_). To achieve this goal, a series of ovule culture experiments were therefore performed with the four flavonoids (ERI, NAR, DHQ and DHK) each at six different concentrations (0, 2, 5, 10, 20 and 40 μM).

**Fig. 2:**
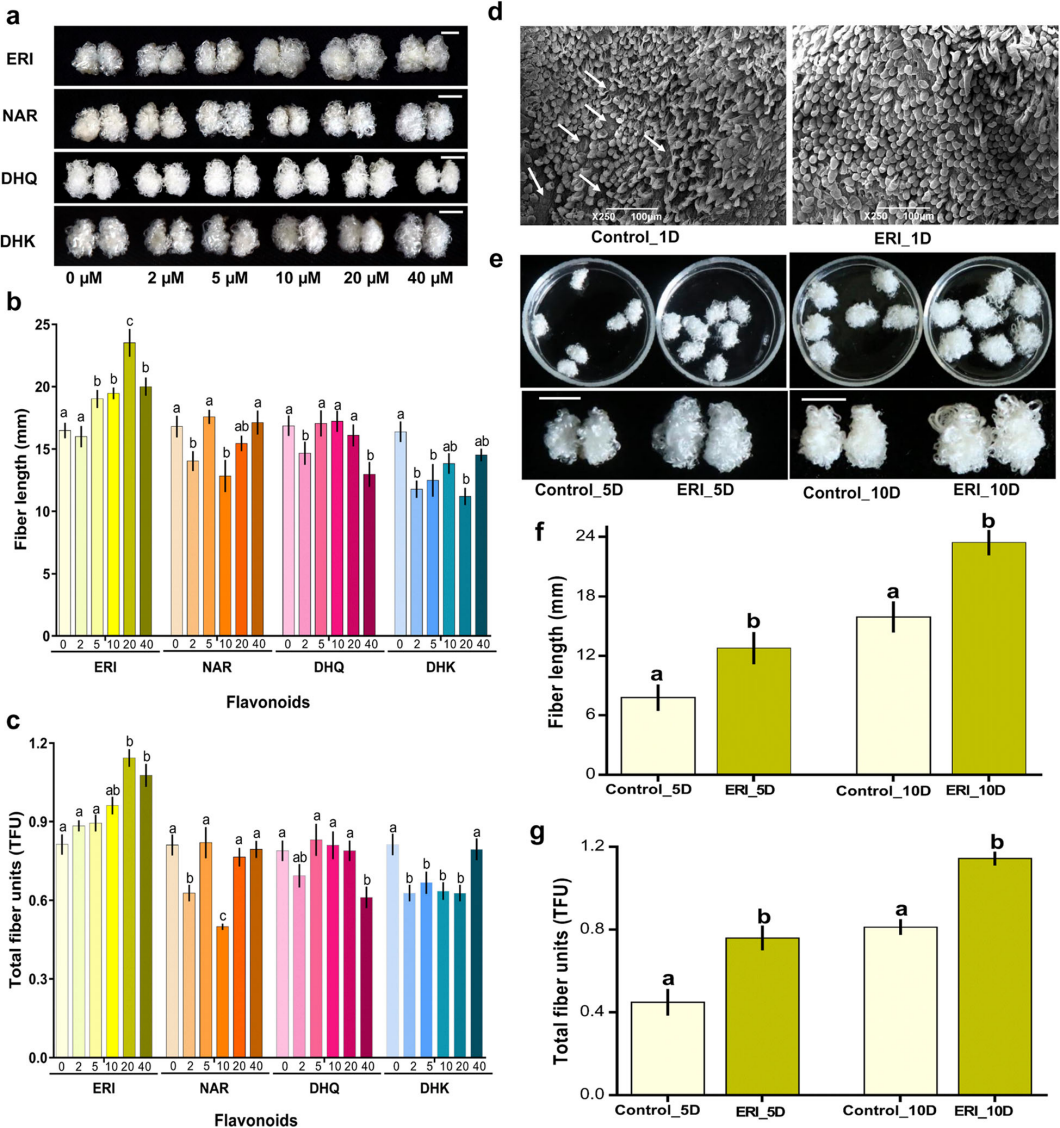
ERI significantly promotes in vitro fibre development. (**a**) The fibre phenotype of ovules cultured in BT medium supplemented with various concentrations (0, 2, 5, 10, 20 and 40 μM) of ERI NAR, DHQ and DHK for 10 days. The white line represents the *scale bar*, 5 mm. (**b**) Fibre length and (**c**) TFU in response to different concentrations of the four flavonoids were plotted as bar plots. For b-c, error bars indicated the SD of 5 biological repeats. Significant differences are presented as lowercase letters. (**d**) Fibre initials were visualized of control and ERI treated ovules after 1 day of incubation using SEM, the s*cale bar* represents 100 μm. (**e**) YZ1 0 DPA ovules were cultured and photographed after 5 and 10 days. For both conditions, the cultured ovules are shown in dishes on the top, while the fibre phenotype of cultured ovules is shown in the lower image, *scale bar*, 5 mm. (**f**) Fibre length and (**g**) TFU were determined in control and ERI treated ovules after 5 and 10 days of culture and are presented as bar plots. Probability values of significant difference based on the Student’s *t test* are indicated

After 10 days of culture, ERI-treated ovules showed more vigorous and fluffier fibre growth when supplemented with 5–40 μM (Fig. 2a). The application of either DHQ or DHK slightly suppressed fibre growth, while no significant differences in fibre development and yield were observed when either 5, 10 or 20 μM concentrations were applied. DHQ inhibited normal fibre development at 2 and 40 μM, while DHK inhibited it at 2, 5 and 20 μM. In contrast ERI showed a concentration-dependent effect on fibre growth. A less consistent pattern was observed for DHK and DHQ, while NAR induced a strong inhibitory effect on fibre length and TFU at 10 μM, but not at lower or higher concentrations (Fig. 2b-c). Fibre growth was not significantly changed after treatment with ERI at 0 μM and 2 μM while it was observed maximum at 20 and 40 μM concentrations (Fig. 2b-c). Overall, ERI significantly promoted the fibre development in terms of length and TFU at 20 μM concentration, which was used for subsequent experiments.

To determine whether ERI has an effect on fibre initiation, ovules cultured for 1 day in BT medium were supplemented with 0 μM (Control_1D) and 20 μM ERI (ERI_1D) and examined for fibre density. Scanning electron microscopic (SEM) images showed normal differentiation and initiation of fibre cells from the ovule epidermal cells in Control_1D, whereas a greater number of initiating fibres and faster elongation were observed in ovule epidermal cells in ERI_1D (Fig. 2d). Therefore, 20 μM ERI application promotes fibre initiation and early elongation within 1 day of application.

Similarly, both early (5 days) and late (10 days) stages of fibre elongation were analyzed for fibre yield to determine whether ERI could significantly affect fibre elongation over a longer developmental period. Ovules showing representative fibre phenotypes from the four treatments (Control_5D, ERI_5D, Control_10D and ERI_10D) are shown in (Fig. 2e). Both fibre length and TFU were significantly enhanced in ERI_5D and ERI_10D ovules compared with Control_5D and Control_10D, respectively (Fig. 2f-g). These findings clearly showed that fibre development was strongly promoted by 20 μM ERI during fibre initiation and early and late fibre elongation stages.

### RNA-seq analysis

To gain insight into transcriptional regulatory networks in response to ERI treatment during initiation and early and late fibre elongation stages, we performed whole transcriptome RNA sequencing analysis for tissues collected from Control and ERI-treated samples after 1, 5 and 10 days (designated as Control_1D, ERI_1D, Control_5D, ERI_5D, Control_10D and ERI_10D respectively). Ovules from 1-day-cultured samples and fibres from 5 and 10-days-cultured samples were collected as three biological replicates, and transcriptome libraries were constructed for 18 samples, i.e., 2 treatments (Control, ERI) each at 3 stages (1, 5, 10 DPA) with 3 replications. Approximately 72.4–74.7 million clean paired-end reads were obtained after trimming adapter sequences and filtering out low-quality reads, and 87.47 - 92.33% reads of these reads were successfully aligned to the *G. hirsutum cv*. TM-1 genome [27]. Of these, a major proportion (60.12 - 67.44%) accounted for uniquely mapped reads, while 22.57 - 29.07% were mapped to multiple sites (Additional file 1: Table S1).

**Fig. 3:**
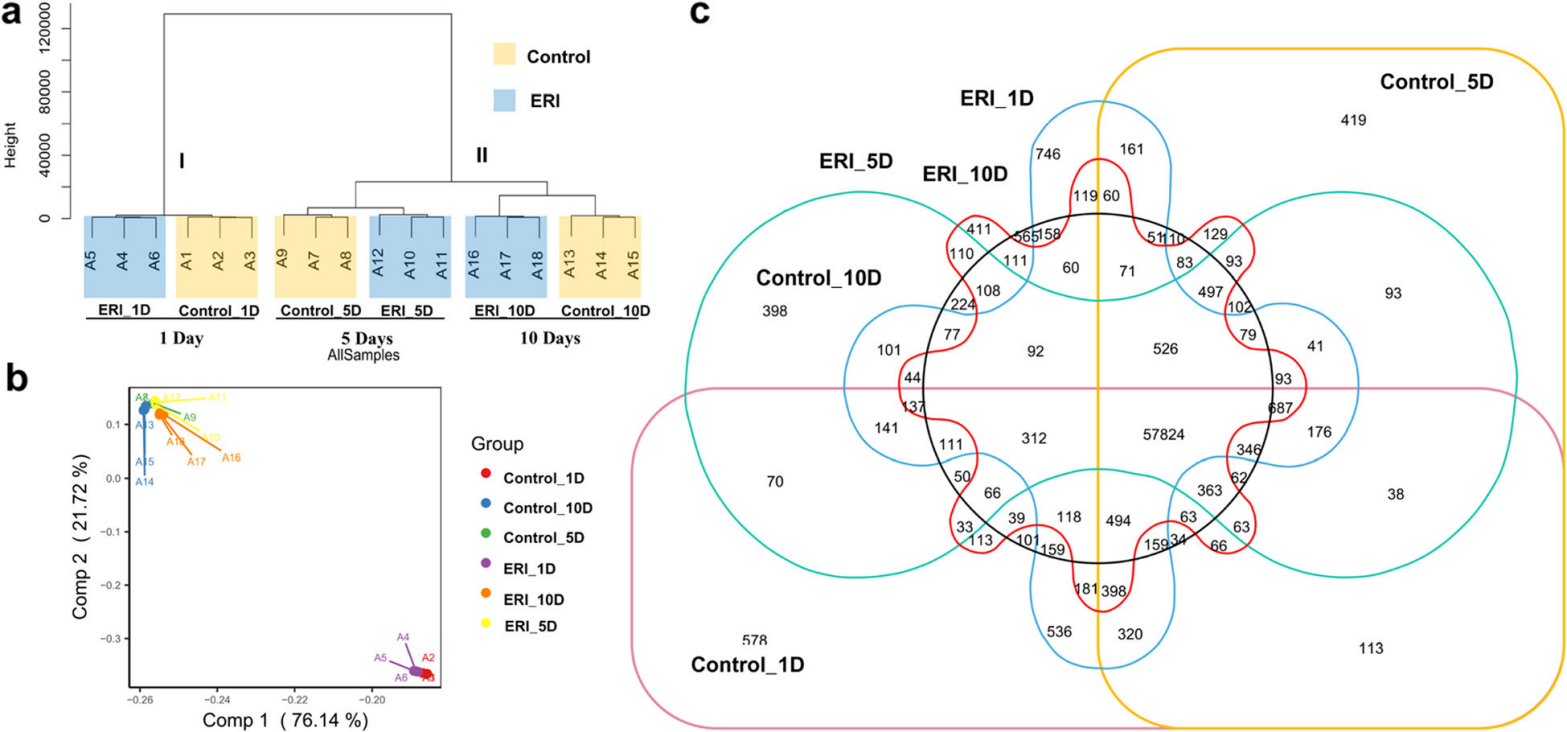
RNA-seq and diversity analysis. (**a**) Cluster diagram generated from 6 RNA-seq datasets. (**b**) PCA plot of 6 RNA-seq datasets from the control and ERI samples. For a-b, each sample consists of three biological repeats, and replications of each treatment are presented with a unique color. (**c**) Venn diagram showing the number of specific genes in the Control_1D, ERI_1D, Control_5D, ERI_5D, Control_10D and ERI_10D

The transcriptional similarity was also ascertained through hierarchical clustering, which distinctively assigned all samples into two major clusters. Cluster I contained replicate samples of Control_1D and ERI_1D while cluster II aggregated all early and late elongation stage samples (Fig. 3a). To minimize the dimensionality in the transcriptomic libraries, the data were normalized to principal components (PCs) through principal component analysis (PCA), and the first two PCs were plotted to visualize variability features. The results showed that samples from the initiation (Control_1D and ERI_1D) and elongation stages (Control_5D, Control_10D, ERI_5D and ERI_10D) were separated from each other (Fig. 3b). Likewise, the expression profiles of 18 samples were well related to the hierarchical clustering results (Fig. 3 a-b; Additional file 2: Figure S1). The results showed that all the samples were clustered according to the distinct biological stages, i.e. initiation and elongation, from which they originated (i.e., 1, 5 and 10 days).

Global transcription profiles were determined for each treatment by analyzing expression data using bioinformatics techniques. The paired comparisons showed that 60,839 to 62,099 genes were co-expressed and 1852 to 2479 genes were specifically expressed in three comparisons (Additional file 3: Figure S2). We found that the majority of the genes were expressed in both the control (59,559 out of 67,713) and ERI (62,071 out of 69,901) in all three treatment samples (Additional file 4: Figure S3). There were 57,824 co-expressed genes among 6 samples, while 38,746 genes were co-expressed in 2 to 5 samples (Fig. 3c). Annotated and novel transcribed regions were identified by the *cuffcompare* utility, which aligns the transcriptome libraries to the TM-1 genome. The results showed that approximately 51,464 to 52,691 annotated genes were identified, i.e. up to 86.98%, while 7712 to 8075 novel transcripts (not annotated) were identified (Additional file 1: Table S1). However, the frequency of expression of annotated and novel transcripts was not significantly different between all Control and ERI paired comparisons (Additional file 5: Figure S4).

### Identification and functional analysis of differentially expressed genes (DEGs)

**Fig. 4:**
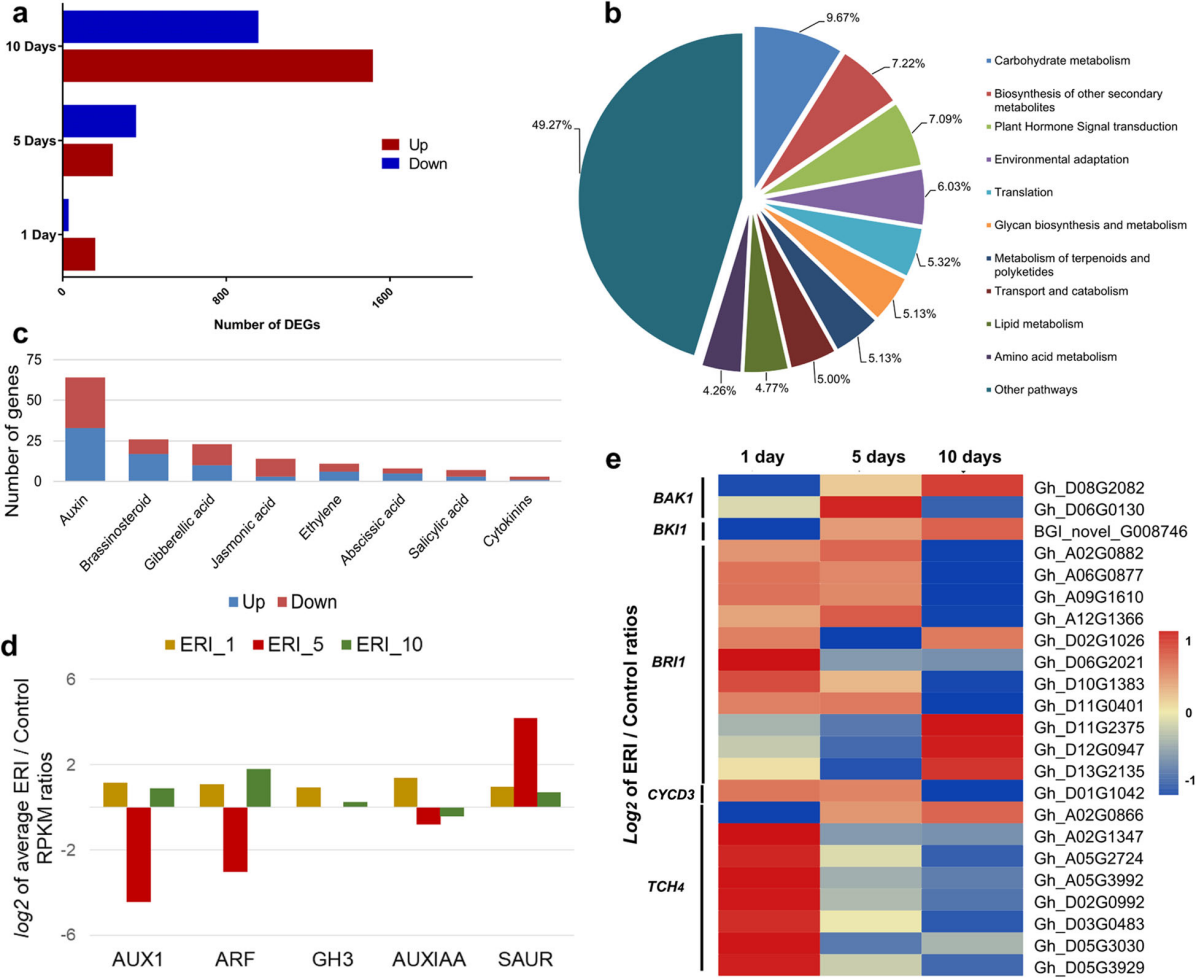
Identification and functional analysis of DEGs. (**a**) DEGs were detected from RNA-seq data of 1, 5 and 10-day-cultured samples in response to ERI treatment. (**b**) The proportions of DEGs enriched in different KEGG annotations included, 10 significantly enriched KEGG pathways (Q value < 0.05). (**c**) The number of DEGs detected in all tissues involved in diverse hormone signaling pathways. (**d**) Expression profiling of differentially expressed auxin signaling pathway genes (*AUX1*, *ARF*, *GH3*, *AUXIAA* and *SAUR*). The *log2* values of ERI/control ratios for all samples, presented as a clustered column chart. (**e**) Heat map of BR signaling pathway genes, shown as the *log2* value of ERI/control ratios

To explore functional aspects of ERI-based fibre elongation, the number of DEGs was detected using criteria of fold change (FC) ratio > 2 and false discovery rate (FDR) < 0.05 to indicate differential expression between control and ERI treatments. Approximately 190 DEGs were detected at fibre initiation (160 up and 30 down regulated between the Control_1D and ERI_1D), 604 at the early elongation stage (245 up and 359 downregulated between the Control_5D and ERI_5D) and 2474 at the late fibre elongation stage (1517 up and 957 down regulated between the Control_10D and ERI_10D; Fig. 4a).

Gene Ontology (GO) and Kyoto Encyclopaedia of Genes and Genomes (KEGG) analysis revealed that the most enriched pathways were associated with carbohydrate metabolism (9.67%), biosynthesis of secondary metabolites (7.22%) and plant hormonal signal transduction (7.09%; Fig. 4b). Among various carbohydrate metabolism pathways, ascorbate and aldarate metabolism are considered critical in elongating fibres, since H_2_O_2_ can act as a cell signaling component, triggering various growth-regulating processes [28]. Elongating cotton fibres exhibit ROS homeostasis [25, 29]. To determine whether ascorbate peroxidases are differentially regulated in response to ERI, *log2* values of ERI/Control ratios were plotted as a heat map (Additional file 6: Figure S5). Our results showed that genes in the ascorbate and aldarate pathway (KEGG00053) were up regulated in ERI_1D (*BGI_novel_G009776* and *Gh_D10G2055*), ERI_5D (*Gh_A02G0706*, *BGI_novel_G000946*, *Gh_D09G0992*, *Gh_A01G2104*, *Gh_D05G0229* and *Gh_D05G3096*) and ERI_10D (*BGI_novel_G004748*, *Gh_A08G1012*, *Gh_A09G1558*, *Gh_D08G0759*, *Gh_D10G1398* and *Gh_D12G1841*) (Additional file 7: Table S2).

Among the phytohormone pathways, auxin and brassinosteroid signal transduction components were significantly upregulated in the ERI-treated samples. Genes in the auxin signaling cascade were most enriched (64 genes), followed by brassinosteroid signaling components (26 genes) (Fig. 4c). Various genes encode proteins involved in the perception of auxin signaling, such as *AUXIN TRANSPORTER PROTEIN1* (*AUX1*), *AUXIN/INDOLE-3-ACETIC ACID* (*AUX/IAA*), *AUXIN RESPONSE FACTORS* (*ARFs*), *GH3* and *SMALL AUXIN UP RNA* (*SAUR*), which showed differential expression patterns in the ERI samples (Additional file 7: Table S2). These auxin signaling genes were upregulated during the initiation (ERI_1) and late elongation (ERI_10) stages, while *AUX1*, *ARF* and *AUXIAA* transcripts were less abundant during the early elongation stage (ERI_5) (Fig. 4d). It was interesting to note that *AUX/IAA*, *GH3* and *SAUR* transcripts, which can regulate auxin-mediated plant growth through cell elongation, were mostly up-regulated in ERI_1D, ERI_5D and ERI_10D (Additional file 7: Table S2).

Brassinosteroids (BRs) are another class of growth promoting hormones, and BR signaling is primarily perceived through *BRASSINOSTEROID INSENSITIVE1* (*BRI1*) and *BCL2-ANTAGONIST/KILLER1* (*BAK1*), which are ultimately transduced to activate expression of genes involved in cell wall modification and cell cycling, such as *XYLOGLUCAN ENDOTRANSGLUCOSYLASE/HYDROLASE* (*TCH4*) and *CYCLIN-D3* (*CYCD3*) [30]. We found that BR biosynthesis pathway genes were up-regulated in response to ERI treatment (Additional file 8: Figure S6). Similarly, some BR signaling cascade components were also found up-regulated in one or more tissues (Fig. 4e). These results suggested that exogenous ERI modulated both auxin and BR biosynthesis and signaling to promote fibre development.

**Fig. 5:**
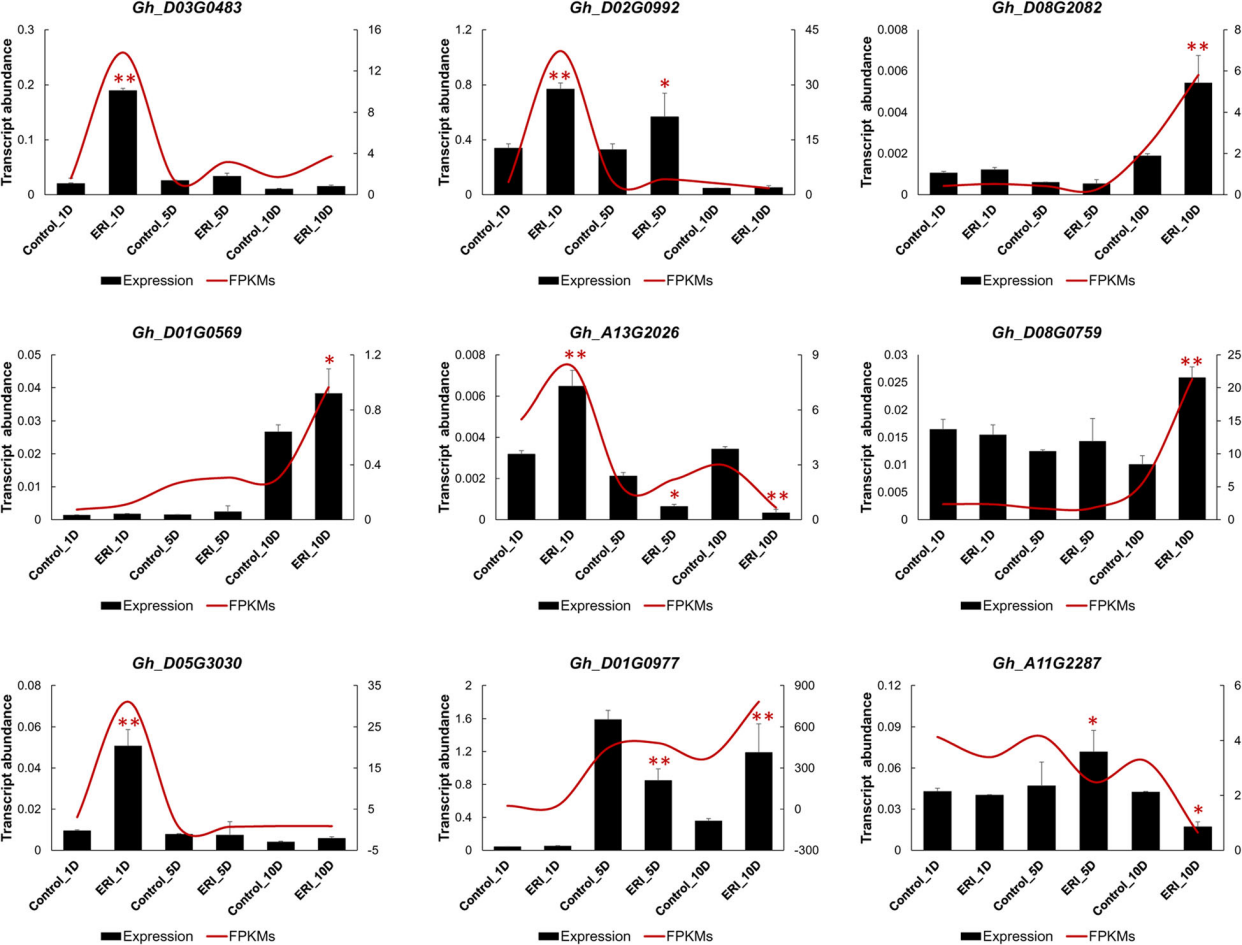
Validation of the expression pattern of some differentially expressed genes. Expression was determined in 6 tissues using RT-qPCR relative to *GhUBQ7*. The SD of three replicates is indicated as error bars. The FPKM values are plotted as a smooth curved red line for comparison. Significant pairwise differences between control and ERI treatment (Control_1D vs ERI_1D, Control_5D vs ERI_5D and Control_10D vs ERI_10D) are denoted as * at 5% and ** at 1%

After determining the KEGG annotations, nine DEGs: *TCH4* (*Gh_D02G0992*, *Gh_D05G3030* and *Gh_D03G0483*), *DFR* (*Gh_D01G0569*), *LAR* (*Gh_D01G0977*), *SAUR* (*Gh_A13G2026*), *ASCORBATE PEROXIDASE* (*APX*; *Gh_D08G0759* and *Gh_A11G2287*) and *BAK1* (*Gh_D08G2082*), were randomly selected to validate the RNA-seq analysis integrity. The RT-qPCR expression was normalized to the expression of the reference gene *GhUBQ7* (NCBI XM_016855110.1). The results showed that the expression profiles of all tested genes were highly related to the fragments per kilo-base of transcript per million mapped reads (FPKMs) values based on the RNA-seq data (Fig. 5). These findings indicated that the results inferred from the RNA-seq data analysis are highly reliable.

### The flavonoid pathway actively modulated in response to ERI

Although other secondary metabolites such as alkaloids and terpenoids have important roles in plants, and are typically species- and tissue-specific, we focused in the current work on flavonoids, since the primary objective of this study was to determine the role of flavonoids and the control of flavonoid biosynthesis in fibre development. Therefore, the expression profiles of flavonoid pathway genes were analyzed. Moreover, we linked flavonoid pathway gene expression to flavonoid content by quantifying flavonoids (ERI, NAR, DHQ, Q, DHK and K) in the six treatment samples.

**Fig. 6:**
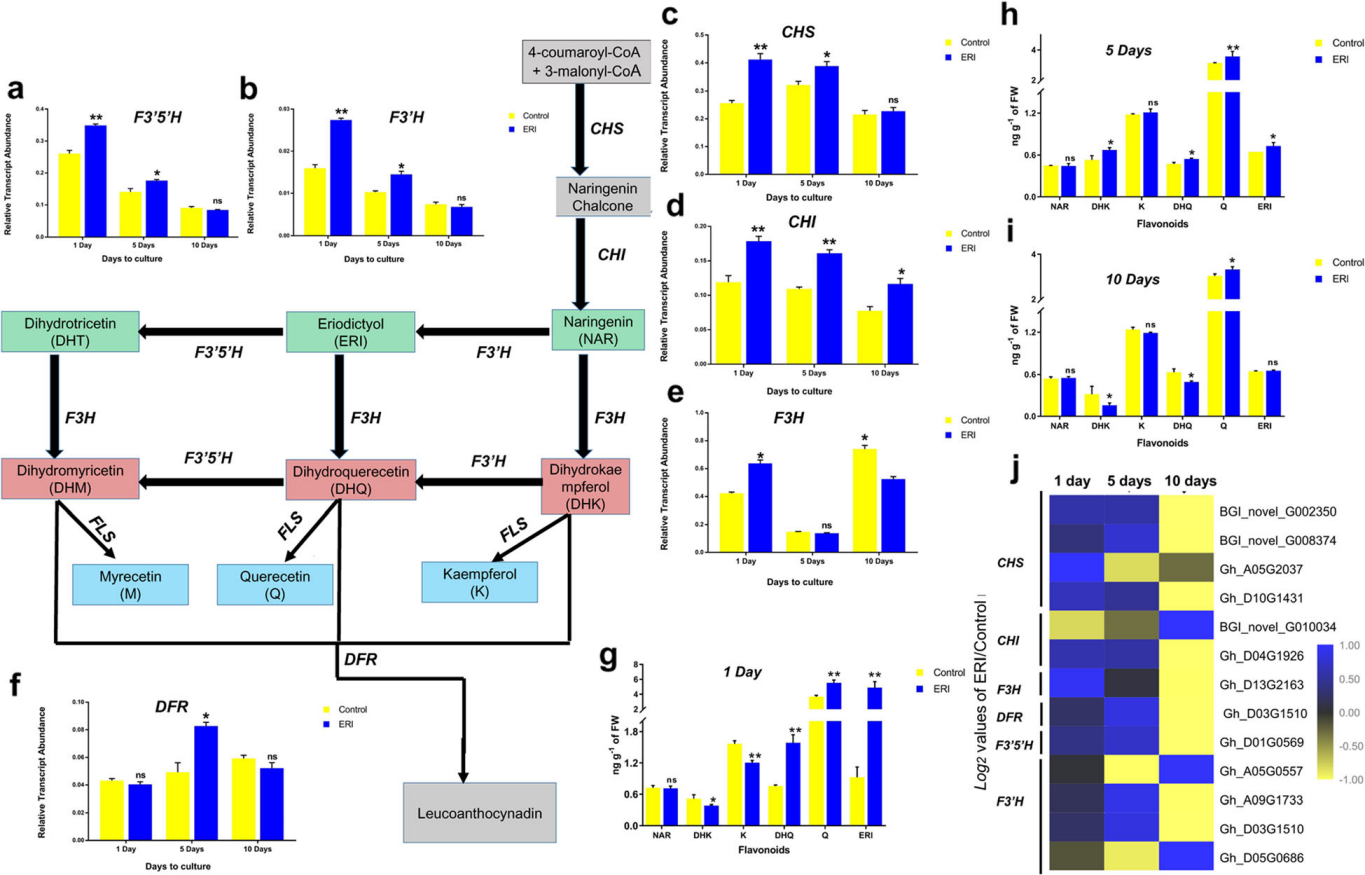
The flavonoid pathway modulated in response to ERI treatment. RT-qPCR analysis of the flavonoid pathway genes (**a**) *F3’5’H*, (**b**) *F3’H*, (**c**) *CHS*, (**d**) *CHI*, (**e**) *F3H*, and (**f**) *DFR* in 1-day ovules and in 5 and 10-day-cultured fibres for the control and ERI treatments. Quantification of different flavonoids (NAR, DHK, K, DHQ, Q and ERI) in Control and ERI samples after (**g**) 1 day, (**h**) 5 days and (**i**) 10 days of culture. For a-i, error bars represent the SD based on three technical repeats. Significant differences were determined and are denoted as * at 5% and ** at 1% significance, while non-significant differences are indicated by ns. (**j**) FPKM values of flavonoid pathway genes derived from RNA-seq analysis are plotted in a heat map as *log2* values of the ERI/control ratios

In the context of flavonoid pathway, the higher *F3’H* transcript abundance might be expected to result in the synthesis of ERI and DHQ through metabolizing NAR and DHK respectively, since the *F3H* transcripts were more abundant in response to exogenous ERI treatment at day 1, ERI_1D (Fig. 6e). Surprisingly, the endogenous NAR content was not significantly lowered in ERI_1D (Fig. 6g) which might be explained by the augmented production of an alternate *F3H* substrate (ERI) or poor relationship between *F3H* transcript and protein. Likewise, it might also be expected because both CHS and CHI were upregulated by ERI treatment (Fig. 6). Moreover, the abundance of *F3’H* transcripts could lead to the catabolism of DHK to DHQ as the endogenous DHQ content was significantly increased, and DHK reduced, even though *F3H* was highly expressed in ERI_1D (Fig. 6a-g). Overall, ERI treatment was associated with the elevated expression of *F3H* and *F3’H* genes and of the upstream flavonoid biosynthesis genes *CHS* and *CHI*.

Despite presumed active biosynthesis of NAR, linked to the up-regulation of *CHS* and *CHI*, it did not accumulate during the initiation and early elongation stages. This may be because the genes capable of metabolizing endogenous NAR were up-regulated in ERI_1D and ERI_5D (Fig. 6a-h). Therefore, DHK tended to accumulate during the early elongation stage (ERI_5D) since *F3H* transcripts were abundant in ERI_1D tissues (Fig. 6a-h). Higher FPKM values for *F3’H* suggested that a higher DHQ content was sustained in ERI_5D fibres, though it did not greatly differ from Control_5D (Fig. 6h). The presence of *F3H* transcripts in early elongation fibre (Control_5D and ERI_5D) may suppress the synthesis of DHQ in ERI_5D (Fig. 6e, h). Moreover, the transcriptional activity of *F3’5’H* may also contributed to the reduced DHQ content in ERI_5D compared with ERI_1D (Fig. 6a, h).

At the maximum fibre elongation stage, flavonoid biosynthesis was suppressed, since *CHS* the transcripts were no longer abundant in ERI_10D fibres (Fig. 6c). The higher *F3H* transcript abundance was associated with significantly higher DHK content in Control_10D (Fig. 6e, i). Even though both *F3H* and *F3’H* were not highly expressed in ERI_10D, ERI content did not change, since it was exogenously applied (Fig. 6b, e-i). To investigate the transcriptional modulation of the flavonoid metabolic pathway in ERI-driven elongated fibre using RNA-seq, FPKM values of putative flavonoid pathway genes were determined and plotted as a heat map (Fig. 6j). All putative *F3’H* transcripts were up-regulated after 1 day in ERI-treated samples, while transcripts of a small number of putative *F3’H* genes were expressed in response to ERI after 5 days (*Gh_A09G1733* and *Gh_D03G1510*) and 10 days (*Gh_A05G0557* and *Gh_D05G0686*) (Fig. 6j). In conclusion, these results show that the expression profiles of flavonoid pathway genes were related to variations in endogenous flavonoid content (Additional file 7: Table S2).

Exogenous supplementation of the flavonoid ERI resulted in the largest number of differentially expressed transcription factors (TFs) in the *MYB* or *MYB*-related category (Additional file 9: Figure S7), and these were differentially expressed in response to ERI (Additional file 10: Figure S8). Our results showed that the expression of putative *β-GALACTOSIDASE* and *XYLOGLUCAN ENDOTRANSGLUCOSYLASE* (*XTH*) genes was also up-regulated in developing fibres in response to ERI (Additional files 11 & 12: Figures S9 & S10; Additional file 7: Table S2). These results showed that key fibre growth-promoting genes were activated by ERI during fibre development.

### ERI and IAA together efficiently promoted cotton fibre development

**Fig. 7:**
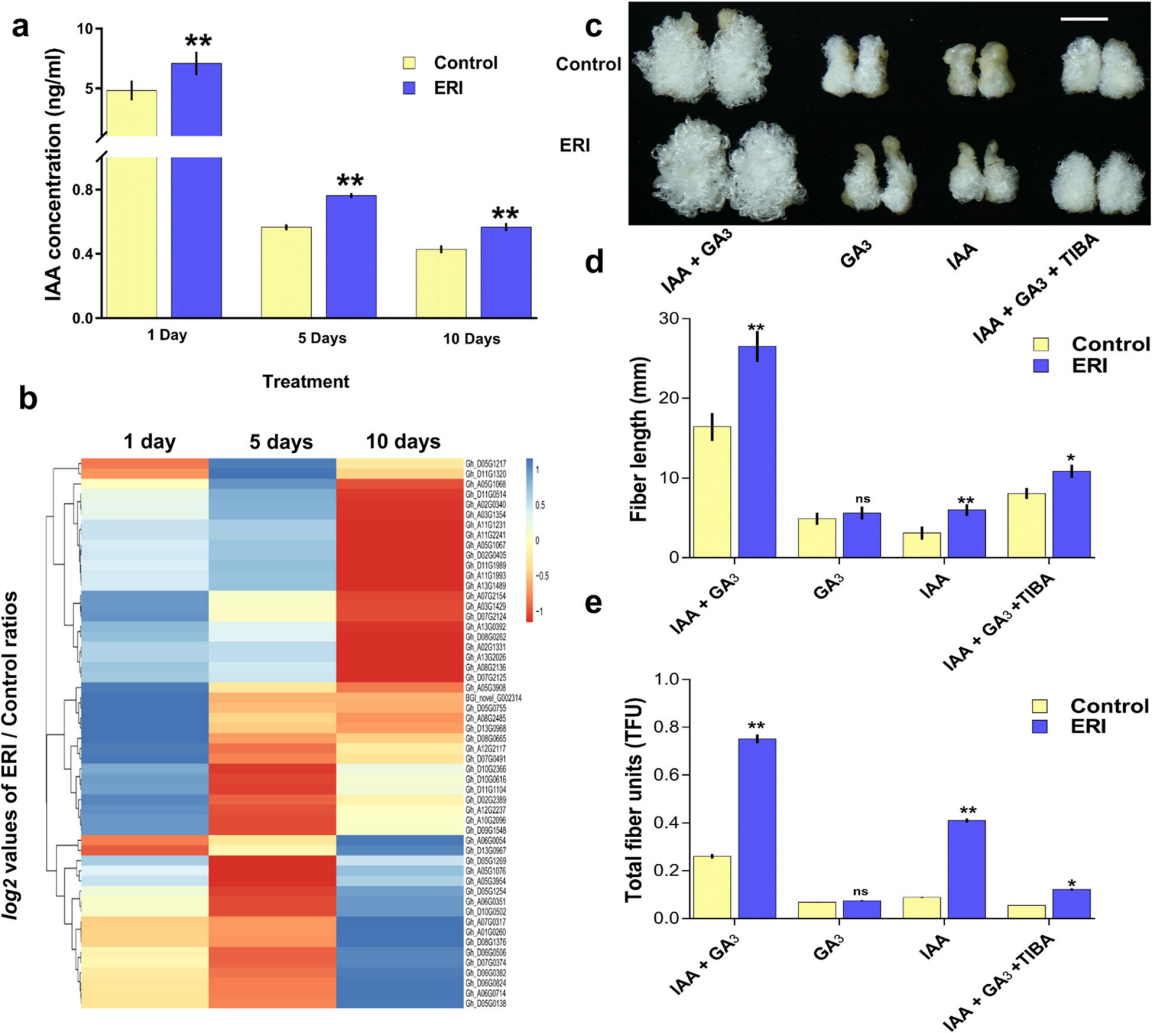
Fibre development was efficiently promoted in the presence of ERI. (**a**) Quantification of IAA in control and ERI treatments after 1, 5 and 10 days. (**b**) *Log*_*2*_ values of the ERI/control ratios for differentially expressed auxin signaling pathway genes (*AUX1*, *AUXIAA*, *GH3* and *SAUR*) based on FPKM values are plotted as a heat map. (**c**) Four different chemical combinations (IAA + GA3, GA_3_, IAA, IAA + GA_3_ + TIBA) were used as treatments in the control (0 μM) and ERI (20 μM). The white line represents the *scale bar*, 10 mm. (**d**) Fibre length and (**e**)TFU were measured in control and ERI ovules after 10 days for the four treatments. Significant differences are denoted as * at 5% and ** at 1% significance, while non-significant differences are indicated by ns

Since both IAA and GA_3_ were supplied in the BT medium for all ovule culture experiments, we tested whether fibre growth could be efficiently improved by ERI application in the presence of IAA or GA_3_ alone. Although RNA-seq based FPKM values suggested that tryptophan (KEGG00380) and diterpenoid biosynthesis (KEGG0904) pathways were differentially regulated (Additional files 13 & 14: Figure S11 & S12), a greater number of auxin signaling genes were up-regulated in response to ERI (Fig. 4c). Moreover, IAA quantification of ovules also revealed that IAA was available at higher concentrations even after 5 and 10 days of culture, i.e., ERI_5D and ERI_10D (Fig. 7a). To gain further insights, the expression profiles of IAA signaling components (*AUX1*, *AUXIAA*, *GH3* and *SAUR*) were determined. The *log*_*2*_ values of ERI/Control ratios of FPKM values were visualized as a heat map, and the auxin signaling genes were found to be differentially expressed in ERI_1D, ERI_5D and ERI_10D (Fig. 7b; Additional file 7: Table S2). These results suggested that activation of the auxin signaling cascade in response to ERI might be potentially involved in the promotion of fibre growth.

Since flavonoid-induced alterations of auxin transport could also lead to temporal auxin accumulation [19], we hypothesized that auxin transport may play a role in determining the ERI-mediated fibre growth response. To test this hypothesis, the auxin transport inhibitor triiodobenzoic acid (TIBA) was added to the BT medium, and interaction effects of both IAA and GA_3_ with ERI were also determined individually. After 10 days, the fibre yield was determined in ovules from four treatments: IAA + GA_3_, GA_3_, IAA and IAA + GA_3_ + TIBA.

Treatment of ovules with either IAA or GA_3_ could not efficiently establish fibre development even in the presence of ERI, showing that both IAA and GA_3_ were required for normal fibre development (Fig. 7c), and the effect of ERI on fibre elongation was not observed in the absence of IAA (Fig. 7c-e). However, ERI with IAA promoted both initiation and elongation even in the absence of GA_3_, though GA_3_ was required for maximum fibre length and TFU (Fig. 7d, e). In summary, ERI and GA_3_ were not sufficient to drive fibre initiation when IAA was not present, while ERI could efficiently promote fibre development in the presence of IAA+ GA_3_ (Fig. 7c-e).

To test our hypothesis concerning whether auxin transport is required for ERI-IAA-driven fibre development, we treated 0 DPA ovules with TIBA in the presence and absence of ERI for 10 days. The results showed that fibre growth was significantly reduced in presence of TIBA (Fig. 7c). However, ERI again demonstrated the potential to positively affect fibre length and TFU even in the presence of TIBA, suggesting the effect of ERI is at least partially independent of polar auxin transport. RNA profiling data supported the view that ERI-induced ROS homeostasis might have contributed to the enhanced fibre growth in the presence of TIBA (Additional file 6: Figure S5). These results demonstrated that ERI may work synergistically with auxin to positively modulate fibre initiation and development.

## Discussion

In the flavonoid metabolic pathway, both NAR and ERI are substrates of *F3H*, which catalyzes the synthesis of dihydroflavanols, and *F3’H* acts on NAR and DHK to produce ERI and DHQ (Fig. 6). The enzyme-substrate interactions have been substantiated through a biochemical analysis of *F3’H* in rice grains [31]. Our results demonstrated that ERI treatment of ovules led to higher *F3H* and *F3’H* transcriptional activities and enhanced expression of the upstream flavonoid biosynthesis genes *CHS* and *CHI*, also linked to higher endogenous ERI content (Fig. 6 a-c). This showed that the flavonoid pathway modulated a complex regulatory network to promote fibre initiation and elongation, which could be triggered by exogenous application of ERI. Our previous study reported that silencing of *F3H* resulted in a short fibre phenotype in transgenic cotton, which could not be rescued in vitro by applying DHK [17]. A second study showed that NAR accumulation suppressed the effect of auxin on fibre development [32]. Therefore the available evidence suggests that NAR acts as negative modulator of cotton fibre development, while ERI acts as a positive modulator of cotton fibre. The transcriptional activity of *F3’H* and its efficient translation into protein could be therefore important in determining fibre quality traits, since it is required for the biosynthesis of both ERI and DHQ (Fig. 6). Therefore, genetic modification of *F3’H*, not *F3H*, could provide enhanced *in planta* fibre growth in cotton, as this is a topic for further study.

A potential role of MYB TFs in regulating fibre development through affecting flavonoid biosynthesis has been previously described [33–35]. Over the past decade, extensive research on fibre biology has revealed many genetic components that are related to enhanced fibre development. For example, xyloglucans play a decisive role in connecting cellulose fibres and cell wall expansion during fibre elongation [36, 37]. Thus, it could be assumed that ERI triggered the fibre elongation mechanism through up-regulating MYB TFs, which somehow up-regulated the putative *β-GALACTOSIDASE* and *XYLOGLUCAN ENDOTRANSGLUCOSYLASE* (*XTH*) genes (Additional files 11 & 12: Figures S9 & S10; Additional file 7: Table S2), and all such factors constituted towards elongated fiber in ERI treated ovules.

The potential involvement of auxin in triggering fibre initiation and modulating fibre development is well established in cotton. The enhanced expression of *iaaM* that is related to auxin production driven by the ovule-specific promoter *FLORAL BINDING PROTEIN7* (*FBP7*) led to increased auxin biosynthesis and significantly promoted fibre yield and quality [32]. There is compelling evidence that flavonoids can regulate auxin signaling and transport directly, *via* influencing the PIN efflux carriers, and auxin biosynthesis indirectly through interactions between auxin and the BR signaling pathways, i.e., through signalling crosstalk between *BRI1* and *ARF* proteins [38]. In the presence of ERI, genes involved in the biosynthetic pathway towards tryptophan, required for auxin production, were up-regulated along with genes involved in auxin signaling cascade. Moreover, IAA levels were shown to accumulate upon ERI treatment (Fig. 7a; Additional file 7: Table S2). Flavonoids may also contribute to fibre elongation through modulating ROS homeostasis, since they act to scavenge ROS [22, 25]. This ROS activity could also determine the concentration of biologically active auxin, as oxidation regulates auxin homeostasis [39]. Linked to this, our results showed that ascorbate peroxidases were up-regulated in ERI-treated ovules and fibres after 1, 5 and 10 days (Additional file 6: Figure S5).

Genetic engineering of the auxin signaling and transportation cascade has aided in promoting fibre development in cotton [40, 41]. Our results further extended the regulatory network of auxin-mediated promoted fibre development by the link with flavonoids such as ERI. Since flavonoid-induced alterations of auxin transport could lead to auxin accumulation [19, 42, 43], we hypothesized that auxin signalling is the mechanism by which ERI-mediated enhanced fibre development is controlled, and our results provided compelling evidences in support of the proposed hypothesis. We therefore concluded that the genetic and metabolic engineering of the flavonoid metabolic pathway could provide opportunities for developing high fibre yielding cotton lines with elite fibre quality parameters, through effects on auxin signalling.

## Conclusion

An integrated approach was adopted to understand the role of flavonoids during cotton fibre development. The outcomes of the study demonstrated that ERI could efficiently promote fibre development. ERI showed a significant dose-dependent effect, with an optimum concentration of 20 μM, in promoting fibre development in terms of length and TFU. Genome wide RNA profiling data indicated that various regulatory pathways like auxin biosynthesis, auxin signalling and transportation along with the ROS homeostasis contributed to the enhanced fibre growth in response to ERI treatment. We also concluded that the flavonoid pathway gene expression was found linked to its content in developing fibres. Moreover, the synergistic action of ERI with auxin might be causative towards positive modulation of in vitro fibre initiation and elongation. Overall, we concluded that the genetic and metabolic engineering of the flavonoid metabolic pathway could provide opportunities for developing high quality cotton fibre.

## Methods

### Plant materials

Seeds of the cotton (*Gossypium hirsutum*) cultivar (YZ1) were collected from the Anyang Cotton Research Institute (CRI), CAAS. The seeds were grown in experimental fields or in greenhouse conditions (28–35 °C day temperature, and 20–25 °C night temperature with + 16/8-h light/dark cycle) at Huazhong Agricultural University, Wuhan, Hubei, China. For the ovule culture assays, flowers were harvested from the same positions on the plant in the afternoon on the day of anthesis (0 DPA) and immediately kept at 4 °C. All chemicals were filter-sterilized, and flavonoids were obtained from Sigma-Aldrich.

### In vitro ovule culture assay and flavonoid treatments

Two hormones, 0.5 μM GA_3_ and 5 μM IAA, were added to BT medium, as the ‘Control’ for all ovule culture experiments. Initially, the exogenous concentration of each of the six flavonoids (ERI, NAR, DHQ and DHK, Q, K) was 10 μM, whereas five concentrations (0, 2, 5, 10, 20 and 40 μM) were used for selected flavonoids in further experiments. The in vitro ovule culture assay was performed following previously described procedures [25]. Briefly, YZ1 flowers were collected from the field or greenhouse in the afternoon at the day of anthesis (0 DPA), and ovaries were excised and surface-sterilized with 0.1% HgCl_2_ for 15 min and rinsed three times with double distilled water (ddH_2_O). Thereafter, ovules from each ovary were dissected using sterilized forceps under aseptic conditions. Approximately 10–15 ovules were suspended in 50 ml control treatment flasks containing flavonoid-supplemented BT medium and, incubated in the dark without agitation at 30 °C for 10 days. Thereafter, fibre-bearing ovules were dried on filter paper. Fibre and ovules were separated in liquid nitrogen, ground into a fine powder and stored at − 80 °C for subsequent analysis.

### RNA-seq analysis and identification of DEGs

Excised 0 DPA YZ1 ovules were cultured in BT medium without (Control) and with ERI treatment (20 μM) for 10 days. The ovule culture assay was performed with three biological repeats along with three technical replicates to minimize experimental error. For genome-wide transcriptome analysis, 1-day control (Control_1D) and ERI (ERI_1D) cultured ovules were dissected under a microscope to collect embryos and immediately stored at − 80 °C Similarly, fibres were separated from 5 and 10 day-cultured samples (Control_5D, Control_10D, ERI_5D and ERI_10D) and immediately stored at − 80 °C.

For transcriptomic library construction, total RNA was extracted from tissues exposed to three treatments with three replicates each (18 samples) as previously described [44]. All samples were collected for RNA-seq by Illumina HiSeq X Ten (BGI, Wuhan, China) and submitted to the NCBI Sequence Read Archive under the Bio project ID PRJNA266265 • SRX4910463 and SRX4910476. Total clean reads were mapped to the *G. hirsutum* (NAU assembly) cotton genome [27] using TopHat2 [45]. The reads overlapping genes were counted using HTseq-count [46]. The counts of genes were used to estimate normalization and dispersions, and they were transformed into variance stabilization data with DESeq [47]. Normalized expression was determined as FPKM (fragments per kilo bases of transcript per million mapped reads) to the reference cotton genome [27], and novel transcripts were determined using *cuff compare*, as described previously [48]. Differentially expressed genes (DEGs) were determined as > 2-fold change (FC) in expression. The digital expression profile of different genes was visualized by plotting the *log2* values of the FPKMs ratio (ERI/Control comparisons) using the Clustvis tool [49].

### RNA extraction and gene expression analysis

For gene expression analysis, fibres and ovules were collected from “Control” and “ERI” cultured samples after 1, 5 and 10 days of culture. Fibre samples were dried on filter paper, ground into a fine powder and immediately stored at − 80 °C for total RNA extraction, as described previously [44]. Briefly, RNA was isolated from all experimental tissues using the Spectrum Plant Total RNA Kit (Sigma-Aldrich, St. Louis, MO). Synthesis of complementary DNA and RT-qPCR were performed as previously described [50]. 3 μg of total RNA was used for double-stranded cDNA synthesis using the reverse transcription enzyme M-MLV (Invitrogen, Super-Script TMII), according to the manufacturer’s instructions.

For the relative quantification of desired genes, RT-qPCR was performed on an ABI Prism 7000 Real-Time PCR system using software (PE Applied Biosystems, USA) according to the manufacturer’s instruction [25]. The RT-PCR mixture was prepared in a final volume of 20 μl containing 0.4 μl of each specific primer (10 μM) and 10 μl of 2 × SYBR Green PCR Master Mix (Applied Biosystems USA). The thermal profile programme was carried out in 96-well optical RT-qPCR reaction plates (Applied Biosystems, USA) under the following conditions: 5 min at 95 °C followed by 30 cycles of 35 s at 95 °C and 35 s at 58 °C. Quantitative reverse transcription RT-qPCR was performed with three biological repeats and at least three technical repeats, while the expression was normalized to *GhUBQ7* and the relative transcript abundance determined using the 2^-∆∆Ct^ method [51]. Primers of flavonoid pathway marker genes and other genes used in the RT-qPCR analysis are listed in Additional file 15: Table S3.

### Quantification of flavonoids and IAA

1, 5 and 10-day-cultured ovules and fibres were washed and dried on filter paper for flavonoid quantification. Ovules from 1 day-cultured ovules, and fibres form 5 and 10 day-cultured ovules were collected from three independent repeats. All samples were ground into powder in liquid nitrogen and 750 μl cold extraction buffer (methanol: water: acetic acid, 80: 19: 1, v/v/v) was added to 0.1 g of sample. Flavonoid quantification was performed according to previous procedures [17]. The flavonoids and IAA quantification were conducted by LC-GC/MS as previously described [52].

### Fibre yield measurement

Fibre yield was determined in terms of fibre length and total fibre units (TFU). For fibre length measurements, fibre-bearing ovules were gently pulled with forceps on filter paper in a straight line. Three biological replicates were assessed, and thirty ovules were used for each replicate. The TFU was measured according to a previously described method with modifications [17]. Briefly, the cultured ovules were washed and boiled in distilled water for 5–7 min until the fibres were dispersed. The ovules were dried on filter paper and stained with 20 ml of 0.02% Toluidine-Blue O solution for 30 s. Next, the ovules were washed with running water for 2–3 min, dried on filter paper and floated in 20 ml glacial acetic acid: ethanol: water (10: 95: 5) solution for 2–4 h. The absorbance was measured in detaining solution at 624 nm with an Infinite 200 PRO multimode reader (Tecan). More than 36 ovules were analyzed in each technical repeat to measure TFU. All experiments were repeated five times, and each replicate constituted at least 6 technical repeats (*n* > 6).

### Scanning electron microscopy (SEM)

For SEM, flowers of YZ1 were picked from the same positions in the afternoon at 0 DPA, and the ovules were cultured for 1 day (24 h) in BT medium with Control and ERI treatments. After 1 day, the ovules were washed with ddH_2_O, immediately fixed in 2.5% glutaraldehyde and stored at 4 °C for 24 h. Thereafter, the samples were rinsed through an ethanol series, transferred to isoamyl-acetate and air dried. Photographs were taken with a JSM-6390/LV SEM (Jeol, Tokyo, Japan) to visualize fibre initials.

### Statistical analysis

All experiments were repeated three times with at least three technical repeats each. For experiments in which more than two treatments were considered, ANOVA and post hoc analysis were performed in Excel to determine statistical significance. For pair-wise comparisons between two treatments, the *Student’s t* test was conducted and significance was determined at 5%.

## Supplementary information

**Additional file 1: Table S1.** Statistics for the paired clean reads and read mapping for 6 treatments with 3 replications.

**Additional file 2: Figure S1.** Bar plots showing the expression distribution pattern in 18 samples.

**Additional file 3: Figure S2.** Venn plots showing pair-wise comparisons in three tissues between control and ERI treatments.

**Additional file 4: Figure S3.** Venn plots showing comparisons of three treatment for both (**a**) control and (**b**) ERI independently.

**Additional file 5: Figure S4.** Comparison of the expression profiles of novel and annotated genes between control and ERI samples.

**Additional file 6: Figure S5.** Heat map showing *log2* values of ERI/control ratios for ascorbate peroxidases.

**Additional file 7: Table S2.** Comparison of the expression profiles of novel and annotated gene between control and ERI samples.

**Additional file 8: Figure S6.** Heat map showing *log2* values of ERI/control ratios for BR biosynthesis pathway genes.

**Additional file 9: Figure S7.** Identification of transcription factors (TFs) that were differentially expressed between control and ERI.

**Additional file 10: Figure S8.** Expression profiles of DEG *MYB* transcription factors presented as log2 values of ERI/Control FPKM ratios.

**Additional file 11: Figure S9.** *Log2* values of ERI/Control FPKM ratios for putative (A) β-galactosidase genes visualized using a heatmap.

**Additional file 12: Figure S10.** *Log2* values of ERI/Control FPKM ratios for putative xyloglucan endotransglucosylase (*XTH*) genes were visualized using a heatmap.

**Additional file 13: Figure S11.** Heat maps showing the expression profiles of IAA (tryptophan) metabolism pathway genes based on *log2* values of ERI/control FPKM ratios.

**Additional file 14: Figure S12.** Heat maps showing the expression profiles of GA_3_ (diterpenoid) biosynthesis pathway genes based on *log2* values of ERI/control FPKM ratios.

**Additional file 15: Table S3.** List of primers used in this study.

## Data Availability

All data files generated by RNA-seq by Illumina HiSeq X Ten (BGI, Wuhan, China) have been submitted to the NCBI Sequence Read Archive under PRJNA266265 • SRX4910463 and SRX4910476.

## Abbreviations

DHK: Dihydrokaempferol and
DHQ: Dihydroquerectin
ERI: Eriodictyol
NAR: Naringenin
SEM: Scanning electron microscopy
TFU: Total fibre units
