# Supplementary material for: Eriodictyol can modulate cellular auxin gradients to efficiently promote in vitro cotton fibre development
Source: BMC Plant Biol. 2019 Oct 24;19:443. doi: 10.1186/s12870-019-2054-x (PMC6814110; doi:10.1186/s12870-019-2054-x)
Supplement: Supplementary file 2 — Additional file 2: Figure S1. Bar plots showing the expression distribution pattern in 18 samples. [file 12870_2019_2054_MOESM2_ESM.docx]

**Figure S1:** Bar plots showing expression distribution pattern in 18 samples.
