# Supplementary material for: Eriodictyol can modulate cellular auxin gradients to efficiently promote in vitro cotton fibre development
Source: BMC Plant Biol. 2019 Oct 24;19:443. doi: 10.1186/s12870-019-2054-x (PMC6814110; doi:10.1186/s12870-019-2054-x)

**Figure S2:** Venn plots showing pair-wise comparisons in three tissues between Control and ERI treatments.


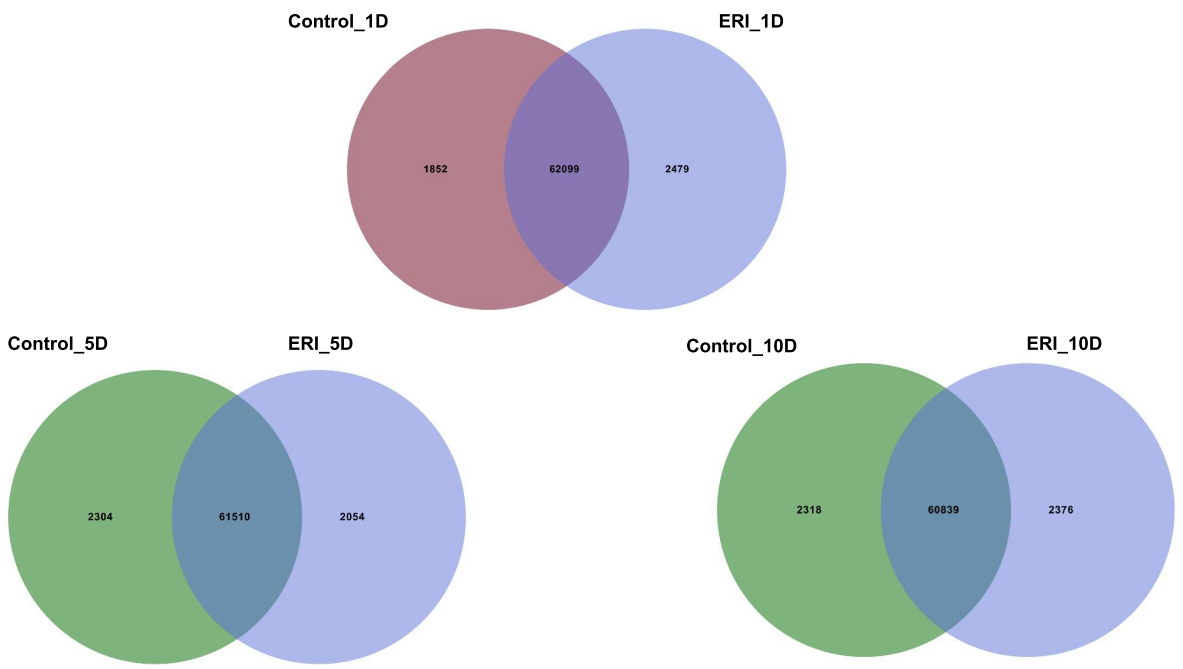

Supplement: Supplementary file 3 — Additional file 3: Figure S2. Venn plots showing pair-wise comparisons in three tissues between control and ERI treatments. [file 12870_2019_2054_MOESM3_ESM.docx]
