# Supplementary material for: Eriodictyol can modulate cellular auxin gradients to efficiently promote in vitro cotton fibre development
Source: BMC Plant Biol. 2019 Oct 24;19:443. doi: 10.1186/s12870-019-2054-x (PMC6814110; doi:10.1186/s12870-019-2054-x)

**Figure S3:** Venn plots showing comparisons of three treatment for both (**A**) Control and (**B**) ERI independently


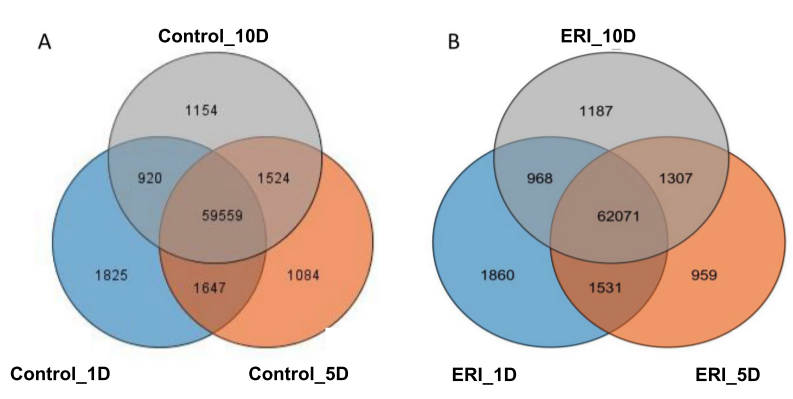

Supplement: Supplementary file 4 — Additional file 4: Figure S3. Venn plots showing comparisons of three treatment for both (a) control and (b) ERI independently. [file 12870_2019_2054_MOESM4_ESM.docx]
