# Supplementary material for: Eriodictyol can modulate cellular auxin gradients to efficiently promote in vitro cotton fibre development
Source: BMC Plant Biol. 2019 Oct 24;19:443. doi: 10.1186/s12870-019-2054-x (PMC6814110; doi:10.1186/s12870-019-2054-x)

**Figure S4:** Comparing expression profiles of Novel and Annotated gene between Control and ERI samples


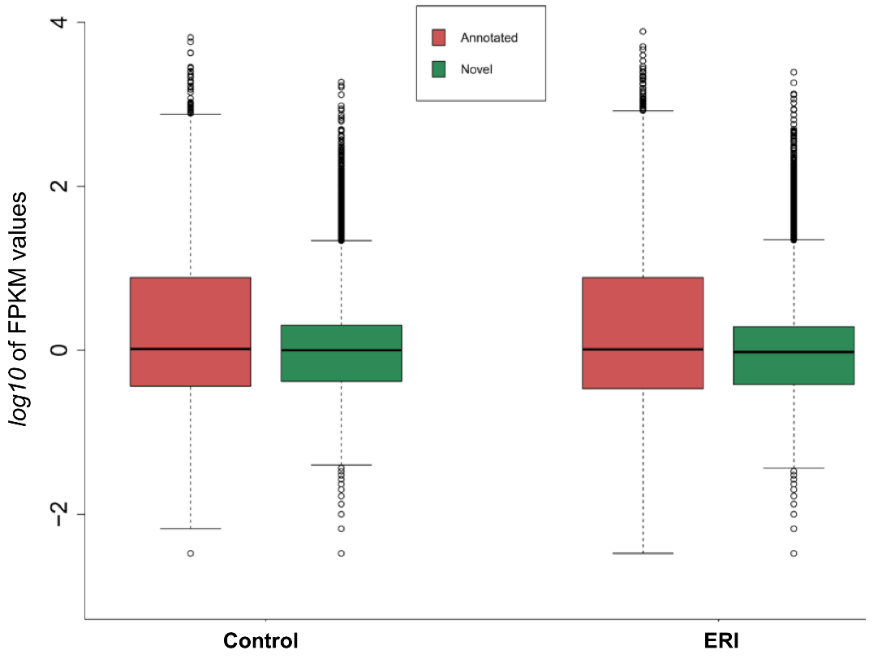

Supplement: Supplementary file 5 — Additional file 5: Figure S4. Comparison of the expression profiles of novel and annotated genes between control and ERI samples. [file 12870_2019_2054_MOESM5_ESM.docx]
