# Supplementary figures and images for: Eriodictyol can modulate cellular auxin gradients to efficiently promote in vitro cotton fibre development
Source: BMC Plant Biol. 2019 Oct 24;19:443. doi: 10.1186/s12870-019-2054-x (PMC6814110; doi:10.1186/s12870-019-2054-x)

**Figure S5:** Heat map showing *log2* values of ERI/Control ratios for ascorbate peroxidases.


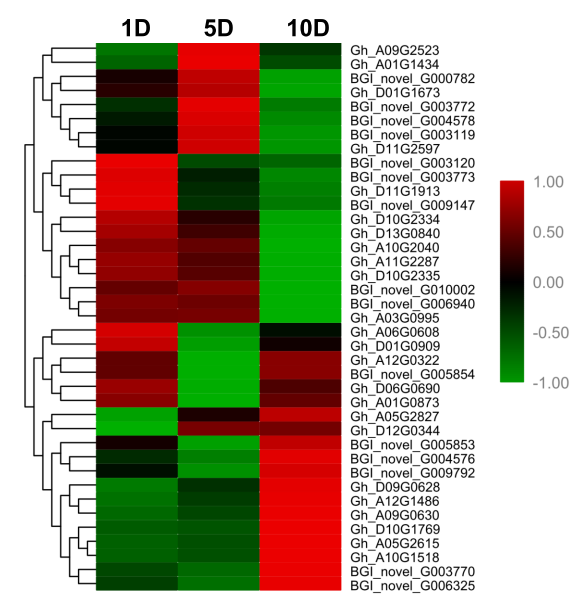

Supplement: Supplementary file 6 — Additional file 6: Figure S5. Heat map showing log2 values of ERI/control ratios for ascorbate peroxidases. [file 12870_2019_2054_MOESM6_ESM.docx]

**Figure S6:** Heat map showing *log2* values of ERI/Control ratios for BR biosynthesis pathway genes.


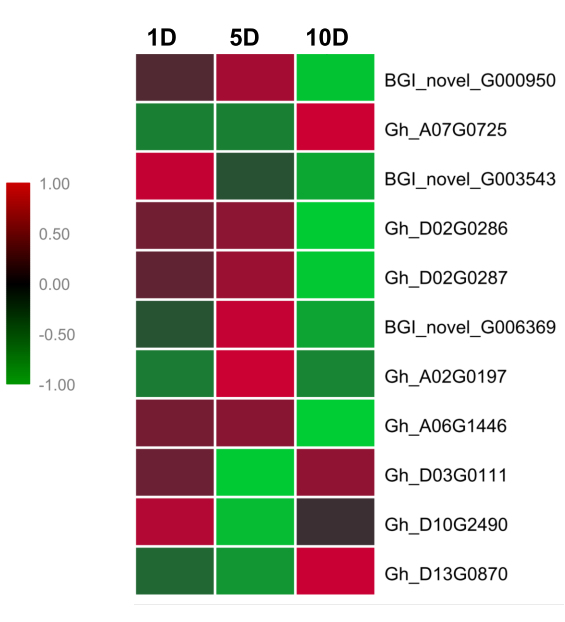

Supplement: Supplementary file 8 — Additional file 8: Figure S6. Heat map showing log2 values of ERI/control ratios for BR biosynthesis pathway genes. [file 12870_2019_2054_MOESM8_ESM.docx]
