# Supplementary material for: Eriodictyol can modulate cellular auxin gradients to efficiently promote in vitro cotton fibre development
Source: BMC Plant Biol. 2019 Oct 24;19:443. doi: 10.1186/s12870-019-2054-x (PMC6814110; doi:10.1186/s12870-019-2054-x)

**Figure S7:** Identification of transcription factors (TFs) differentially expressed between Control and ERI.


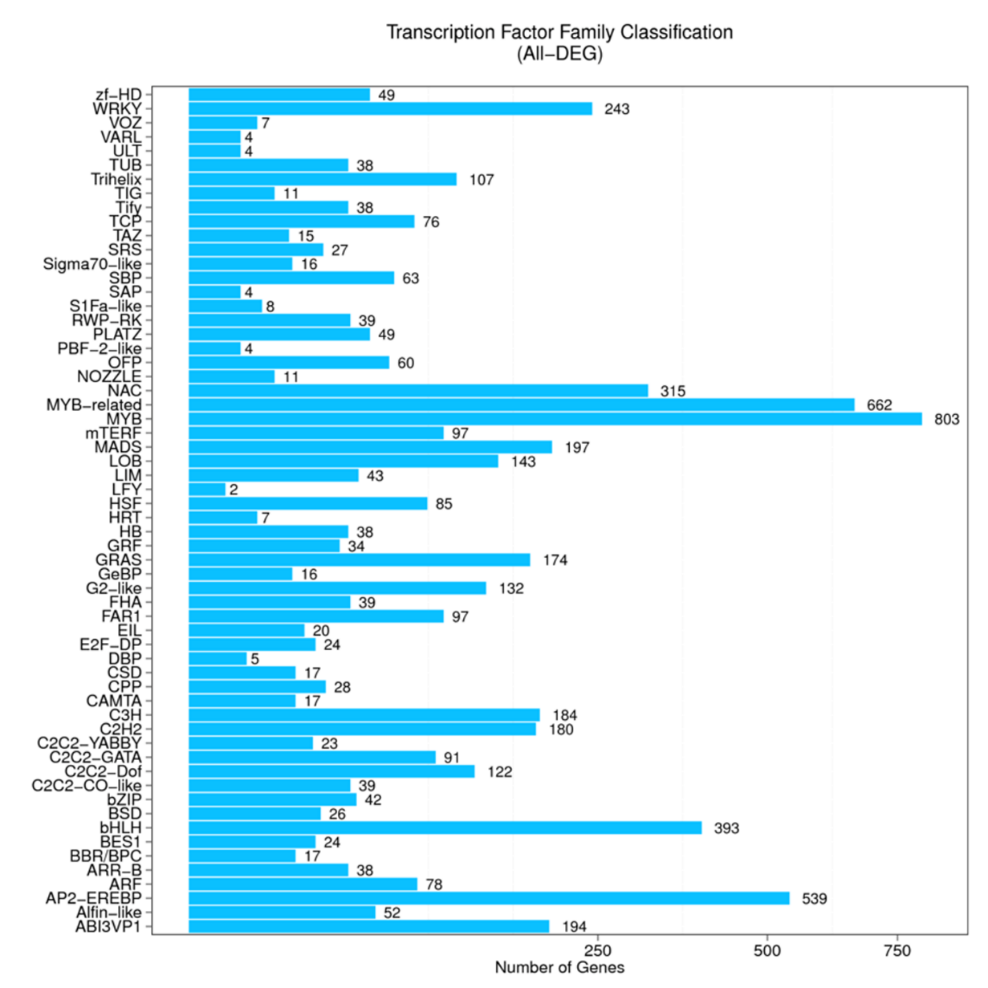

Supplement: Supplementary file 9 — Additional file 9: Figure S7. Identification of transcription factors (TFs) that were differentially expressed between control and ERI. [file 12870_2019_2054_MOESM9_ESM.docx]
