# Supplementary material for: Eriodictyol can modulate cellular auxin gradients to efficiently promote in vitro cotton fibre development
Source: BMC Plant Biol. 2019 Oct 24;19:443. doi: 10.1186/s12870-019-2054-x (PMC6814110; doi:10.1186/s12870-019-2054-x)

**Figure S8:** Expression profiles of DEG *MYB* transcription factors presented as log2 values of ERI/Control FPKM ratios.


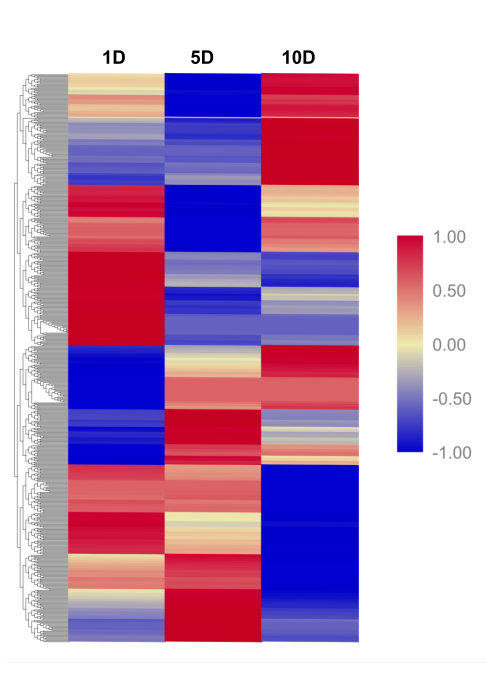

Supplement: Supplementary file 10 — Additional file 10: Figure S8. Expression profiles of DEG MYB transcription factors presented as log2 values of ERI/Control FPKM ratios. [file 12870_2019_2054_MOESM10_ESM.docx]
