# Supplementary material for: Eriodictyol can modulate cellular auxin gradients to efficiently promote in vitro cotton fibre development
Source: BMC Plant Biol. 2019 Oct 24;19:443. doi: 10.1186/s12870-019-2054-x (PMC6814110; doi:10.1186/s12870-019-2054-x)

**Figure S9:** *Log2* values of ERI/Control FPKM ratios for putative (A) β-galactosidase genes were visualized through heatmap.


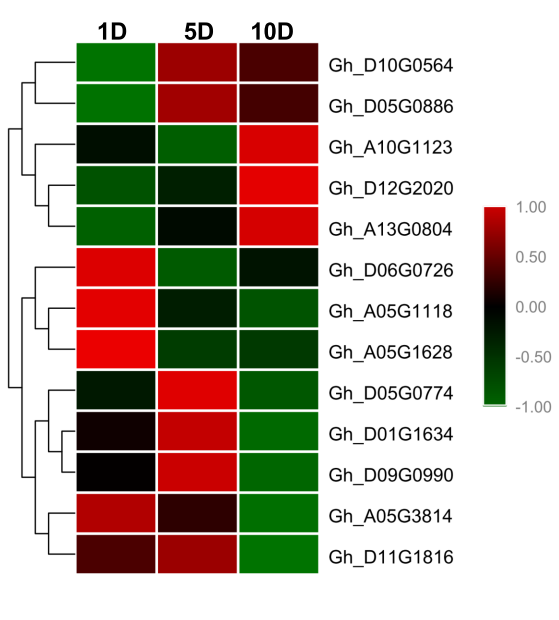

Supplement: Supplementary file 11 — Additional file 11: Figure S9. Log2 values of ERI/Control FPKM ratios for putative (A) β-galactosidase genes visualized using a heatmap. [file 12870_2019_2054_MOESM11_ESM.docx]
