# Supplementary material for: Eriodictyol can modulate cellular auxin gradients to efficiently promote in vitro cotton fibre development
Source: BMC Plant Biol. 2019 Oct 24;19:443. doi: 10.1186/s12870-019-2054-x (PMC6814110; doi:10.1186/s12870-019-2054-x)

**Figure S10:** *Log2* values of ERI/Control FPKM ratios for putative xyloglucan endotransglucosylase (*XTH*) genes were visualized through heatmap.


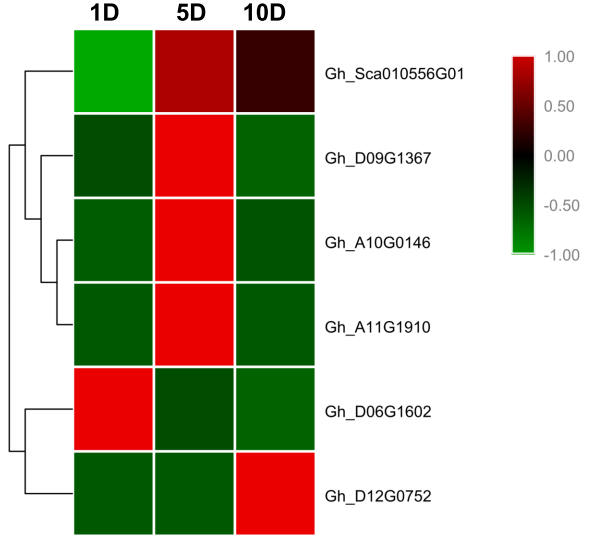

Supplement: Supplementary file 12 — Additional file 12: Figure S10. Log2 values of ERI/Control FPKM ratios for putative xyloglucan endotransglucosylase (XTH) genes were visualized using a heatmap. [file 12870_2019_2054_MOESM12_ESM.docx]
