# Supplementary material for: Eriodictyol can modulate cellular auxin gradients to efficiently promote in vitro cotton fibre development
Source: BMC Plant Biol. 2019 Oct 24;19:443. doi: 10.1186/s12870-019-2054-x (PMC6814110; doi:10.1186/s12870-019-2054-x)

**Figure S11:** Heat maps showing expression profiles of IAA (tryptophan) metabolism pathway genes based on *log2* values of ERI/Control FPKM ratios.


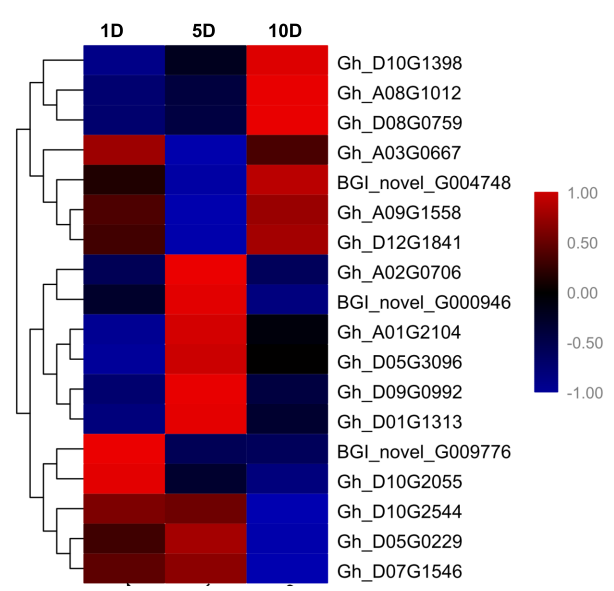

Supplement: Supplementary file 13 — Additional file 13: Figure S11. Heat maps showing the expression profiles of IAA (tryptophan) metabolism pathway genes based on log2 values of ERI/control FPKM ratios. [file 12870_2019_2054_MOESM13_ESM.docx]
