# Supplementary material for: Eriodictyol can modulate cellular auxin gradients to efficiently promote in vitro cotton fibre development
Source: BMC Plant Biol. 2019 Oct 24;19:443. doi: 10.1186/s12870-019-2054-x (PMC6814110; doi:10.1186/s12870-019-2054-x)

**Figure S12:** Heat maps showing expression profiles of GA_3_ (diterpenoid) biosynthesis pathway genes based on *log2* values of ERI/Control FPKM ratios.


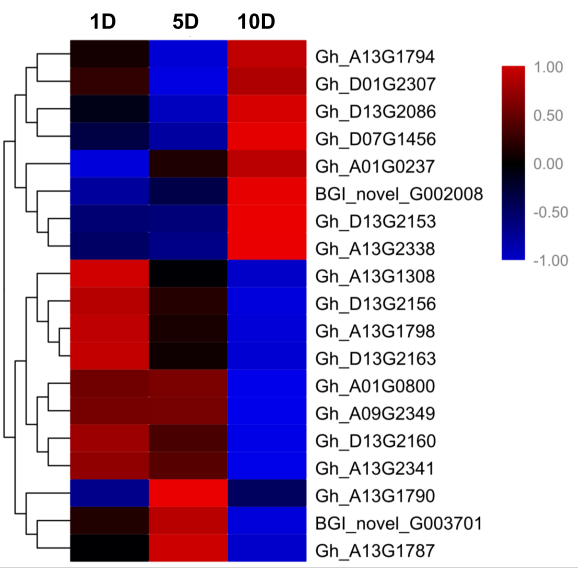

Supplement: Supplementary file 14 — Additional file 14: Figure S12. Heat maps showing the expression profiles of GA3 (diterpenoid) biosynthesis pathway genes based on log2 values of ERI/control FPKM ratios. [file 12870_2019_2054_MOESM14_ESM.docx]
